# The impact of emotional value in digital teaching resources on learning motivation—an empirical analysis based on a questionnaire survey

**DOI:** 10.3389/fpsyg.2026.1733324

**Published:** 2026-03-24

**Authors:** Wenping Xu, Peipei Hu, Yongzhou Zhu, Yalan Chen, Tongtong Cheng, Chao Liu, Leiming Mao, Aiming Shen, Xiaojin Cui

**Affiliations:** 1 Department of Medical Technology, Nantong Health Vocational College, Nantong, China; 2 Department of Medical Informatics, School of Medicine, Nantong University, Nantong, China; 3 School of Medical Technology, Jiangsu College of Nursing, Huai’an, China; 4 Nantong Mental Health Center, Nantong Fourth People's Hospital, Nantong, Jiangsu, China

**Keywords:** digital teaching resources, emotionally empowering value, learning motivation, platform empowerment, self-empowerment

## Abstract

**Background:**

The rapid expansion of digital teaching resources under initiatives such as Smart Education and Internet + Education has provided higher vocational education with unprecedented access to diverse platforms and instructional tools. However, increased accessibility has not produced proportional gains in learning motivation, creating a paradox of “more resources, weaker motivation.” Understanding the motivational mechanisms behind this imbalance has become a pressing challenge.

**Objective:**

This study explores how emotionally empowering value embedded in digital teaching resources affects students’ learning motivation and identifies the emotional dimensions and pathways through which empowerment exerts its effects.

**Methods:**

A structured questionnaire was administered to 1,303 medical students in five-year higher vocational programs. The survey comprised demographics and usage patterns, a 26-item Emotionally Empowering Value Scale (six dimensions), and a 13-item Learning Motivation Scale grounded in the Attention, Relevance, Confidence, and Satisfaction model (ARCS). Data were analyzed using SPSS 25.0 and Python via descriptive statistics, Pearson correlations, and multiple regression.

**Results:**

Students exhibited relatively high learning motivation, with significant variations by gender, grade, and resource-use frequency. All six emotionally empowering value dimensions were positively associated with the three motivation subdimensions (all *p* < 0.001), with strong relationships overall. Classroom empowerment showed the strongest correlation with Learning Interest (*r* = 0.95), while platform empowerment also demonstrated strong associations with Learning Interest (*r* = 0.92) and Persistence and Confidence (*r* = 0.90). In the dual-pathway regression models, both platform empowerment and self-empowerment were significantly associated with motivational outcomes (platform *β* = 0.632–0.676; self *β* = 0.269–0.315; all *p* < 0.001), highlighting consistent patterns across outcomes within this sample.

**Conclusion:**

Embedding emotional value within digital teaching resources is essential for sustaining students’ motivation. Platform empowerment enhances persistence and confidence through external feedback, while self-empowerment fosters intrinsic engagement and self-directed learning. These findings extend Self-Determination Theory and the ARCS model into digital learning contexts and offer practical guidance for designing emotionally supportive learning environments in higher vocational education.

## 1 Introduction

With the rapid advancement of information technology, digital teaching resources have become a crucial supporting force in driving the reform of higher vocational education (Cerino et al., 2025; Ning et al., 2025). Under the broader “Smart Education” and “Internet + Education” agenda, online course platforms, educational apps, and digital instructional videos continue to expand, providing abundant content and flexible learning pathways (Jain et al., 2025; Qian et al., 2025). However, a noteworthy phenomenon persists: despite improvements in accessibility and technical capabilities, students’ learning motivation does not necessarily increase accordingly (Liu et al., 2025; Sanri, 2025; van Dijk et al., 2025), and some learners even exhibit the paradox of “more resources, weaker motivation” (Japelj Pavešić et al., 2021). Recent work examining learning behaviors on digital platforms suggests that motivation and knowledge development are shaped not only by resource availability, but also by platform-level learning processes and feedback/analytics mechanisms, which help explain why “more” does not necessarily mean “more motivating” (Noor et al., 2022; Younas et al., 2022). This gap between resource abundance and motivational outcomes motivates a closer examination of what makes digital resources genuinely motivating.

Learning motivation is a core psychological variable that shapes learners’ willingness to participate, persist, and complete learning tasks (Shahrokhi and Dehaghani, 2025; Shekarchizadeh et al., 2025). According to Ryan and Deci’s Self-Determination Theory (SDT), motivation depends not only on task appeal but also on learners’ supportive perceptions of the environment, particularly relatedness (emotional support), competence, and autonomy (Ahumada-Newhart and Eccles, 2020; Grabowski et al., 2021). In AI-driven digital transformation contexts, students’ technological self-efficacy and perceived support from digitally mediated teaching practices can function as key enabling conditions for satisfaction of autonomy and competence needs, thereby influencing motivational outcomes in online environments (Younas et al., 2026; Younas et al., 2024). In traditional face-to-face instruction, teachers’ facial expressions, vocal tones, and immediate feedback often provide emotional support that strengthens goal identification and belongingness (Muñoz-Troncoso and Riquelme, 2025; Wang et al., 2025). By contrast, the decontextualized and low-interaction nature of many digital environments may weaken or even negate this emotional support mechanism (Miller-Graff et al., 2024; Neudert et al., 2023). Therefore, embedding emotional value into digital teaching resources is likely to be critical for stimulating and sustaining learning motivation.

Recent work has begun to address this challenge through concepts such as Emotionally Adaptive Design (Bermudez et al., 2019; Rivet et al., 2023), Immersive Experience (Dhar et al., 2021; Hazarika and Rahmati 2023), and Human-Computer Affective Interaction (Castiblanco Jimenez et al., 2024; He, 2023). Meanwhile, emerging evidence links emotionally supportive digital learning experiences to students’ behavior, well-being, and resilience (Younas et al., 2025b). Building on this line of work, a recent review of AI-driven approaches to self-directed learning further highlights how AI-enhanced environments can scaffold learners’ autonomy and engagement—pointing to the need to connect affective design with motivational mechanisms and analytics-informed evaluation (Younas et al., 2025a). Preliminary evidence indicates that digital resources incorporating positive feedback, life relevance, and emotional resonance can better stimulate learning interest and sustain motivation (Kikuchi, 2025; Le et al., 2022). Taken together, these studies suggest that motivational gains depend on whether digital ecosystems combine affective support with autonomy/competence scaffolding and analytics-informed feedback, rather than on resource expansion alone. However, a clear research gap remains: existing studies often emphasize design principles or general user perceptions, while systematic empirical investigations that operationalize emotional value as a multidimensional construct and test how different dimensions translate into motivation—especially in higher vocational learning contexts—are still scarce. In particular, evidence remains limited on which specific components of emotionally empowering value most strongly relate to different facets of motivation.

Meanwhile, national and regional initiatives have substantially strengthened the supply of digital resources. Platforms such as the Smart Education of China Platform and MOOC have aggregated large quantities of digital educational resources (Lee and Song, 2022; Xu et al., 2025; Wu et al., 2024), and institutionally developed high-quality courses and virtual simulation training systems have supported five-year higher vocational education reforms in Jiangsu Province (Wang et al., 2023). Digital textbooks that integrate lesson plans, core materials, and supplementary resources into “learning resource packages” further expand access and content diversity (Jang et al., 2025; Peterson et al., 2004). Yet large-scale resource construction and integration alone do not guarantee motivation; motivational effectiveness likely depends on whether these resources provide emotional scaffolding that supports learners’ engagement and psychological needs.

Learning engagement reflects sustained affective and cognitive activity (Hampton et al., 2020; Lees-Murdock et al., 2024). Positive emotional experiences such as focus, enjoyment, and satisfaction can facilitate cognitive development and strengthen motivation (Li et al., 2023; Donnelly et al., 2020), and successful experiences in digital environments—such as completing virtual experiments or receiving feedback—may further enhance motivation and interest (Karayilan et al., 2022). Emotion also directly influences attention, memory retention, and mental agility, thereby shaping learning efficacy (Li et al., 2022; Sewell et al., 2020). However, many digital resources still overemphasize technical functionality while underemphasizing affective design, resulting in depersonalized learning experiences (Guo et al., 2022). Furthermore, standardized designs may fail to accommodate individual differences in affective needs (Lang et al., 2022). These considerations reinforce the need to examine emotional value in digital teaching resources using a clear, testable framework.

Accordingly, this study focuses on five-year higher vocational students and establishes an analytical framework with “Emotionally Empowering Value” as the independent variable and “Learning Motivation” as the dependent variable. Three questions are addressed: (1) Which dimensions of emotional value significantly influence motivation? (2) What are the relative effects of platform empowerment versus self-empowerment? (3) How can digital resource design be optimized based on empirical evidence? By quantifying emotionally empowering value and comparing platform versus self-empowerment, this study provides actionable evidence for improving digital teaching resource design in higher vocational education. Through questionnaire surveys and multiple regression analysis, this study provides both empirical evidence and design implications for creating warmer, motivationally supportive digital learning environments.

## 2 Research methodology

### 2.1 Research design

This study employed a quantitative, cross-sectional design using a structured questionnaire survey to examine the effects of emotionally empowering value in digital teaching resources on students’ learning motivation. The research framework was theoretically grounded in SDT and Keller’s Attention, Relevance, Confidence, and Satisfaction model (ARCS) of Motivational Design, which jointly informed the conceptual pathways through which affective empowerment shapes motivational outcomes (Keller, 1987).

The questionnaire was developed through literature synthesis, expert consultation, and pilot testing to ensure linguistic clarity and structural coherence. All scale items adopted a 5-point Likert rating system (1 = Strongly Disagree, 5 = Strongly Agree), ensuring consistency in response interpretation and cross-variable comparability.

### 2.2 Participants and sampling strategy

The study targeted students enrolled in five-year higher vocational medical programs in Jiangsu Province, including Nursing, Pharmacy, and Medical Laboratory Technology majors. A hybrid cluster-convenience sampling approach was adopted, with classroom-based paper questionnaires distributed by course instructors. Specifically, the sample was drawn from one institution using classroom clusters across three majors (Nursing, Pharmacy, and Medical Laboratory Technology) and four cohorts (Grades 2021–2024). Instructors distributed paper questionnaires using standardized instructions, emphasizing anonymity and that participation would not affect course evaluation or grades.

A total of 1,307 questionnaires were collected, of which 1,303 met the inclusion criterion of a ≥90% valid completion rate for Likert-scale items (Q18–Q56), equivalent to at least 35 valid responses. The effective recovery rate was 96.5%.

Within the valid sample, females constituted 82.8% and males 17.2%. Participants’ ages ranged from 14 to 24 years (*M* = 17.28, SD = 1.20). Most participants were aged 17–22, with a small number of younger/older students due to the five-year vocational track and individual enrollment variations. Grade distribution was dominated by sophomores (27.4%) and juniors (24.9%), with primary specializations in Nursing (39.4%), Pharmacy (34.6%), and Medical Laboratory Technology (24.3%). The sample size and coverage across cohorts and majors provided a reasonable basis for subsequent inferential analysis.

### 2.3 Research instruments and variable architecture

The questionnaire consisted of three sections totaling 56 items (details in the Supplementary Material 1).

Section 1: Demographic Profile and Digital Resource Usage (Items 1–17). This section assessed participants’ basic information—age, gender, grade level, and major—as well as digital resource use patterns, including frequency of engagement (single-response), platform preferences (multiple-response), and learning purposes (multiple-response). The use of multi-response items aimed to capture a more comprehensive picture of learners’ backgrounds and digital exposure contexts.

Section 2: Emotionally Empowering Value Scale (Items 18–43). This section was derived from SDT-informed external support mechanisms and prior research on affective engagement in gamified learning (Alsawaier, 2018). It operationalized six empowerment dimensions aligned with the analytical framework used in the results: ① Self-Empowerment (Items 18–22): Measures autonomous exploration, learning agency, and confidence cultivation during resource utilization; ② Teacher Empowerment (Items 23–26): Evaluates instructional support through pedagogical interactivity, feedback quality and expressive scaffolding; ③ Peer Empowerment (Items 27–30): Assesses collaborative engagement, belongingness reinforcement, and community co-creation experiences; ④ Family Empowerment (Items 31–34): Examines the home learning environments, emotional affirmation and proactive parental attention; ⑤ Platform Empowerment (Items 35–39): Quantifies interface intuitiveness, feedback responsiveness, operational efficiency and resource diversity; ⑥ Classroom Interaction Empowerment (Items 40–43): Captures task challenge calibration, situational immersion, and expressive freedom within classroom-based learning designs.

All items were rated on a 5-point Likert scale (1 = Strongly Disagree, 5 = Strongly Agree), reflecting the perceived intensity of emotionally empowering value within each context. Conceptually, the interpersonal facets (teacher, peer, and family empowerment) map onto SDT need-supportive experiences (e.g., relatedness support and autonomy−/competence-supportive cues), while platform/classroom empowerment captures environmental affordances that may enable such need satisfaction in digitally supported learning.

Section 3: Learning Motivation Scale (Items 44–56). Anchored in Keller’s ARCS model, this 13-item section assessed three subdimensions consistent with the subsequent Results analysis: ① Learning Interest (Attention Sustenance and Exploratory Drive; Items Q44–Q46): Assess the resource’s capacity to capture and sustain students’ interest while stimulating knowledge-seeking initiative; ② Learning Relevance (Goal Value and Alignment; Items Q47–Q49): Gauge learners’ perceived identification with instructional objectives and recognition of pragmatic significance; ③ Persistence and Confidence (Learning Continuity and Confidence; Items Q50–Q56): Measure students’ task-specific confidence, sustained learning intent, and persistence in completing learning activities.

Instrument validation: The final survey instrument underwent expert review by five domain experts in educational psychology and medical pedagogy to evaluate content relevance and construct adequacy. A pilot test (*n* = 60) verified item wording and scale clarity and showed high internal consistency (Cronbach’s *α* = 0.94).

### 2.4 Data processing procedures

The collected data underwent a series of cleaning, validation, and inferential analyses as described below. All data were processed using SPSS 25.0 and Python 3.10. Questionnaires with less than 90% completion were excluded, resulting in 1,303 valid samples for analysis. Reliability was assessed via Cronbach’s α. Across the six empowerment dimensions and three motivational subdimensions, α coefficients were consistently high (α range 0.941–0.981). To provide validity evidence beyond Cronbach’s α, we additionally examined composite reliability and convergent validity (CR range 0.943–0.982; AVE range 0.819–0.923 across constructs). Exploratory factorability checks indicated excellent sampling adequacy and significant Bartlett’s tests. Specifically, for empowerment items (Q18–Q43), KMO = 0.980 and Bartlett’s test was significant [*χ*^2^(325) = 56061.93, *p* < 0.001]; for motivation items (Q44–Q56), KMO = 0.975 and Bartlett’s test was significant [*χ*^2^(78) = 30663.46, *p* < 0.001]. Given the single-source self-report design, we also conducted a common-method check (Harman’s single-factor test) on the core items (Q18–Q56), where the first unrotated factor accounted for 78.78% of the variance, indicating a strong general factor and motivating cautious interpretation of association magnitudes.

Chi-square tests (*χ*^2^) were employed to examine group differences in motivation tiers across gender, grade, program type and frequency of digital resource engagement. Effect sizes were quantified using Cramér’s *V*. Pearson correlation analysis explored associations between the six dimensions of Emotionally Empowering Value and the three subdimensions of Learning Motivation based on mean composite scores of Likert-scale items.

Finally, multiple linear regression analyses were conducted to identify key predictors of learning motivation. The six empowerment dimensions served as independent variables, and the three motivational subdimensions-Learning Interest, Learning Relevance, and Persistence and Confidence-as dependent variables. A dual-pathway regression model incorporating Platform Empowerment (external support) and Self-Empowerment (internal drive) was further tested. Model fit was evaluated using *R*^2^, standardized *β* coefficients, and significance levels (*p* < 0.05), and residual and fitted-value diagnostics were used to check general model assumptions. Given the high intercorrelations among empowerment facets, multicollinearity diagnostics were examined and the regression coefficients were interpreted as relative indicators under substantial shared variance rather than as fully independent effects. Additional EFA details and discriminant validity checks are provided in Supplementary Text 1.

## 3 Results

### 3.1 Classification of learning motivation levels and sample characteristics

This study employed a criterion-referenced approach to classify learning motivation levels. Specifically, based on the mean scores of Items Q44–Q56 (“Learning Motivation Assessment”) in the questionnaire, the 5-point Likert scale responses were stratified into five tiers: ≥4.5: “Very High”; 4.0–4.49: “High”; 3.0–3.99: “Moderate”; 2.0–2.99: “Low”; <2.0: “Very Low.” This classification integrates conventional Likert scale interpretation guidelines with theoretical demarcations from SDT and the ARCS Model of Motivational Design, enabling differentiation of student cohorts across distinct motivational manifestation states (Table 1).

**Table 1:** Classification and interpretation of learning motivation levels based on SDT and ARCS models.

Motivation level	Score range (5-point)	SDT motivation type	ARCS model manifestation
Very high	4.5–5.0	Intrinsic motivation/Integrated regulation	High interest, sustained goal commitment, high satisfaction
High	4.0–4.49	Identified regulation	Strong task value perception
Moderate	3.0–3.99	External regulation (controlled)	Task completion with limited engagement
Low	2.0–2.99	Amotivation/external control	Distracted attention, low persistence
Very low	<2.0	Amotivation	Goal absence, absence of self-driven action

SDT, self-determination theory; ARCS, attention, relevance, confidence, and satisfaction.

Based on the above classification criteria, statistical analysis of 1,303 valid questionnaire samples found: Participants’ ages were mainly concentrated between 17 and 22 years old, with a mean age of 17.28 years (SD = 1.20). Females accounted for 82.8% of the sample, males for 17.2%. Regarding digital teaching resource usage, students’ frequency of use was generally high, with the combined proportion of “3–5 times per week” and “daily use” exceeding 60%. Commonly used platforms included instructional videos, online course platforms, and educational apps. Usage purposes primarily focused on “supplementing classroom knowledge”, “completing learning tasks” and “expanding knowledge scope” etc.

### 3.2 Distribution of learning motivation levels across demographic characteristics

**Figure 1:**
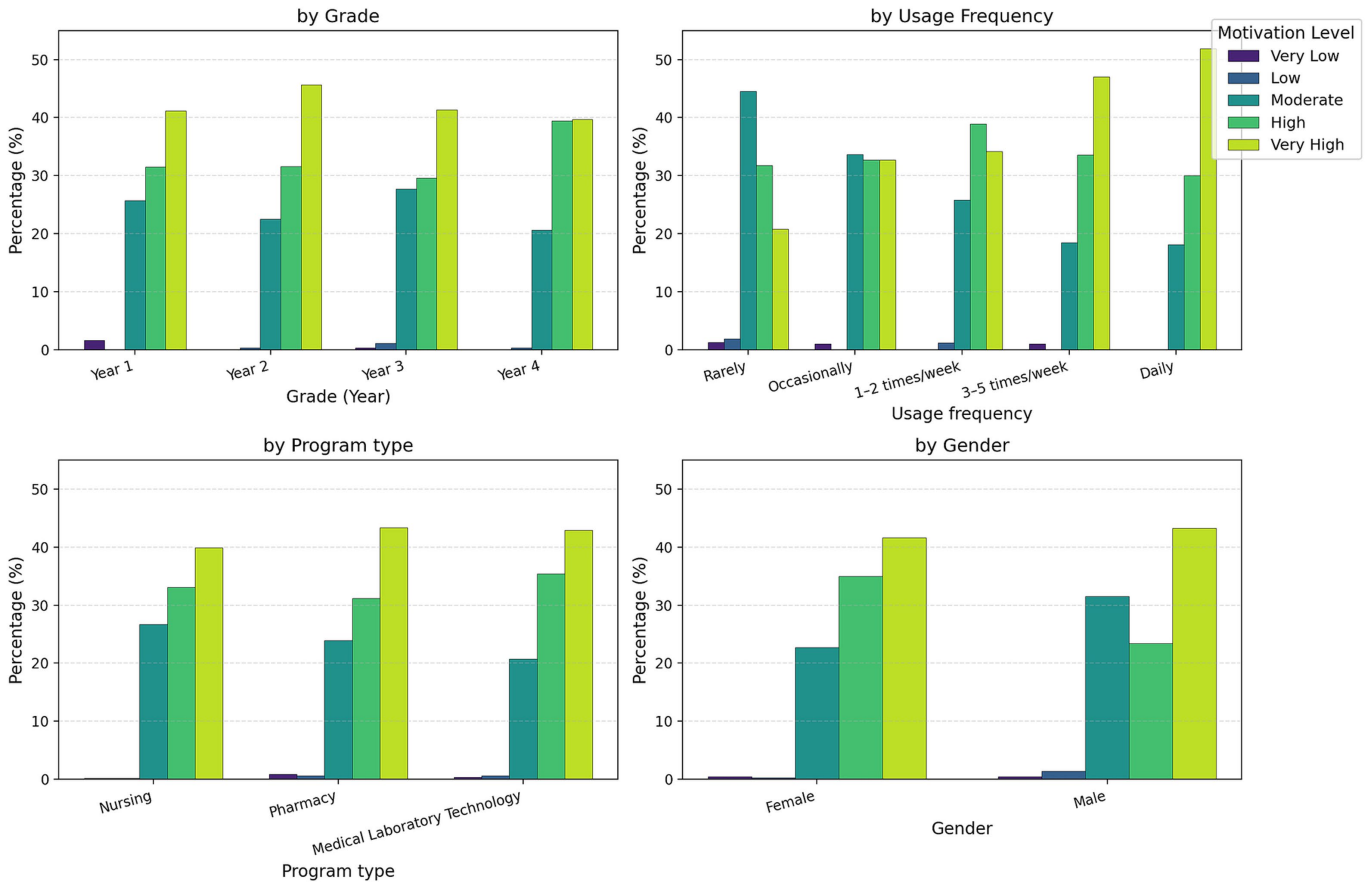
Distribution of learning motivation levels across demographic characteristics. *χ*^2^ tests revealed significant associations for gender, grade, and usage frequency (*p* < 0.05), with usage frequency showing the strongest effect. Motivation levels are ordered from very low to very high.

To investigate the influence of demographic variables on learning motivation levels, this study utilized gender, grade level, program type, and digital resource engagement frequency as independent variables. We examined distributional differences in learning motivation tiers (Very High, High, Moderate, Low, and Very Low) across variable groupings, with statistical significance assessed via chi-square tests (Figure 1).

Regarding gender differences, female students exhibited a “Very High” motivation level proportion of 41.6%, with “High” at 34.9%, collectively accounting for 76.5%. Among male students, the “Very High” proportion was 43.2%-slightly exceeding female-while the “High” motivation proportion decreased to 23.4%, and “Moderate” motivation increased to 31.5%. The association between gender and motivation levels was statistically significant [*χ*^2^ (4) = 18.04, *p* = 0.001, Cramér’s *V* = 0.12], indicating structural differences in learning motivation manifestations across genders.

Regarding grade-level trends, learning motivation differed across grades. Year 4 students demonstrated a combined “Very High” and “High” motivation proportion of 79.1%, compared to 70.9% among Year 3 students. Chi-square testing confirmed that grade-level differences in motivation tiers were statistically significant [*χ*^2^(12) = 28.37, *p* = 0.005, Cramér’s *V* = 0.09], indicating a plausible moderating effect of grade level on motivation intensity.

Regarding program types, motivational tier distributions varied across disciplinary cohorts. For instance, certain programs exhibited “Very High” motivation proportions exceeding 43%, while others demonstrated relatively higher proportions of “Moderate” motivation levels. However, the association between program type and motivation tiers did not reach statistical significance [*χ*^2^(8) = 8.60, *p* = 0.377, Cramér’s *V* = 0.06].

Regarding the usage frequency variable, it showed the most significant differences. Among students who used digital resources daily, the proportion of ‘Very High’ motivation reached 51.9%, while among low-frequency users, the proportion of ‘Moderate’ and lower motivation levels significantly increased. The association between this variable and motivation levels was highly significant [*χ*^2^(16) = 105.58, *p* < 0.001, Cramér’s *V* = 0.14], supporting the hypothesis that ‘higher usage frequency leads to stronger learning motivation’, which aligns with the theoretical connotations of the “Relevance” and “Satisfaction” dimensions in the ARCS model.

In summary, distributional differences across learning motivation tiers demonstrated statistical significance for gender, grade level, and resource engagement frequency, with usage frequency exhibiting the comparatively strongest effect. These findings provide theoretical grounding and actionable guidelines for designing tiered pedagogical interventions and evidence-based resource optimization strategies aligned with motivational hierarchies.

### 3.3 Correlation analysis between emotionally empowering value dimensions and learning motivation

**Figure 2:**
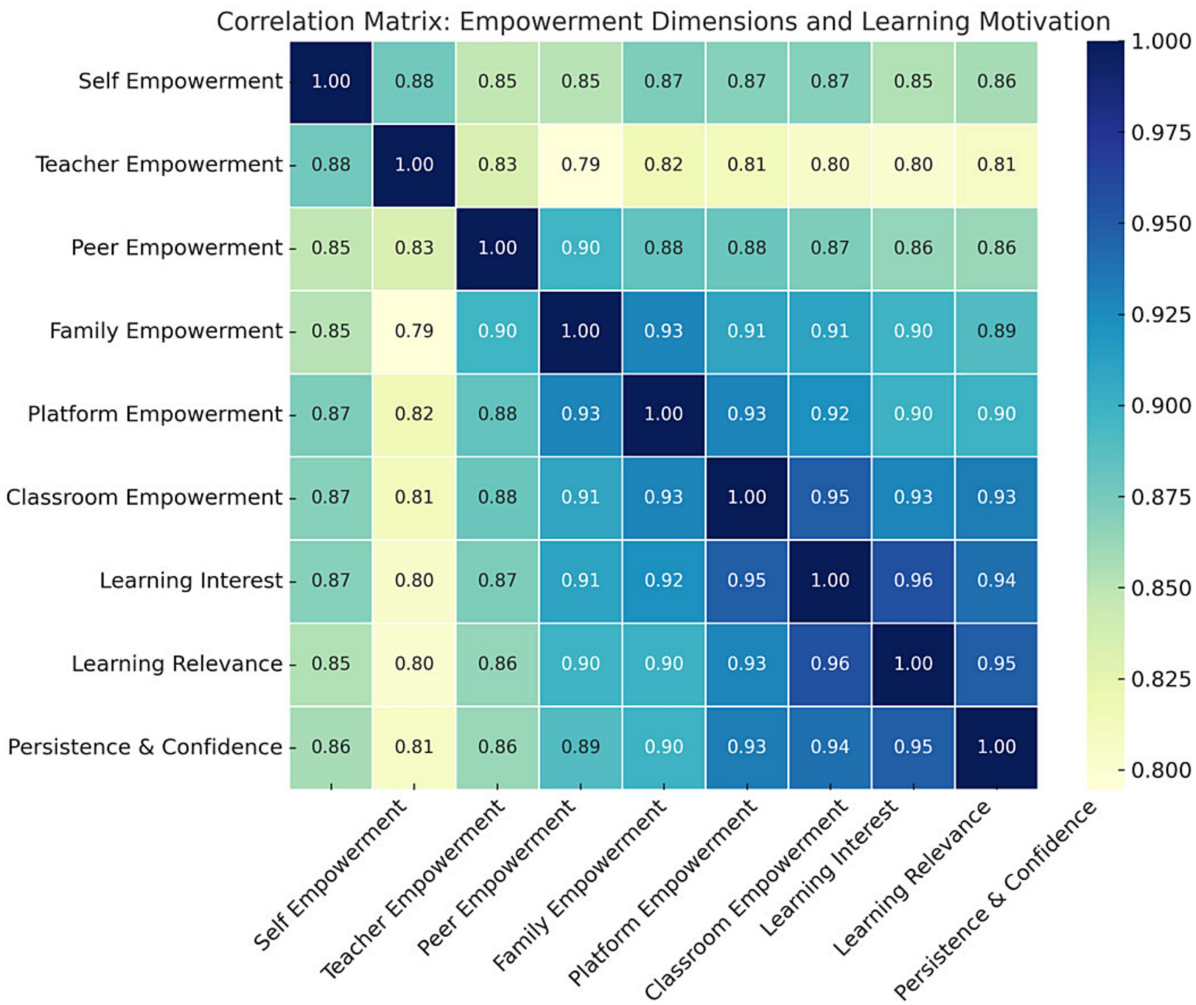
Correlation analysis between emotionally empowering value dimensions and learning motivation. All correlations were significant at *p* < 0.001.

This study revealed statistically significant and strong positive correlations between all six emotionally empowering value dimensions and the three learning motivation subdimensions (Figure 2; all *p* < 0.001). Overall, the associations were strong, which is plausible given that the empowerment dimensions represent closely related supportive perceptions within a broader emotionally empowering value framework, and motivation subdimensions are also conceptually adjacent. Notably, classroom empowerment demonstrated the strongest association with Learning Interest (*r* = 0.95) and remained strongly related to Learning Relevance (*r* = 0.93) and Persistence and Confidence (*r* = 0.93). Platform empowerment also showed strong associations with Learning Interest (*r* = 0.92) and Persistence and Confidence (*r* = 0.90), followed by self-empowerment (*r* = 0.87 and *r* = 0.86, respectively). Given the single-survey self-report design, these correlation magnitudes should be interpreted cautiously, and subsequent regression analyses focus on relative patterns rather than causal inference.

### 3.4 Analysis of key predictive factors across emotionally empowering value dimensions on learning motivation

**Figure 3:**
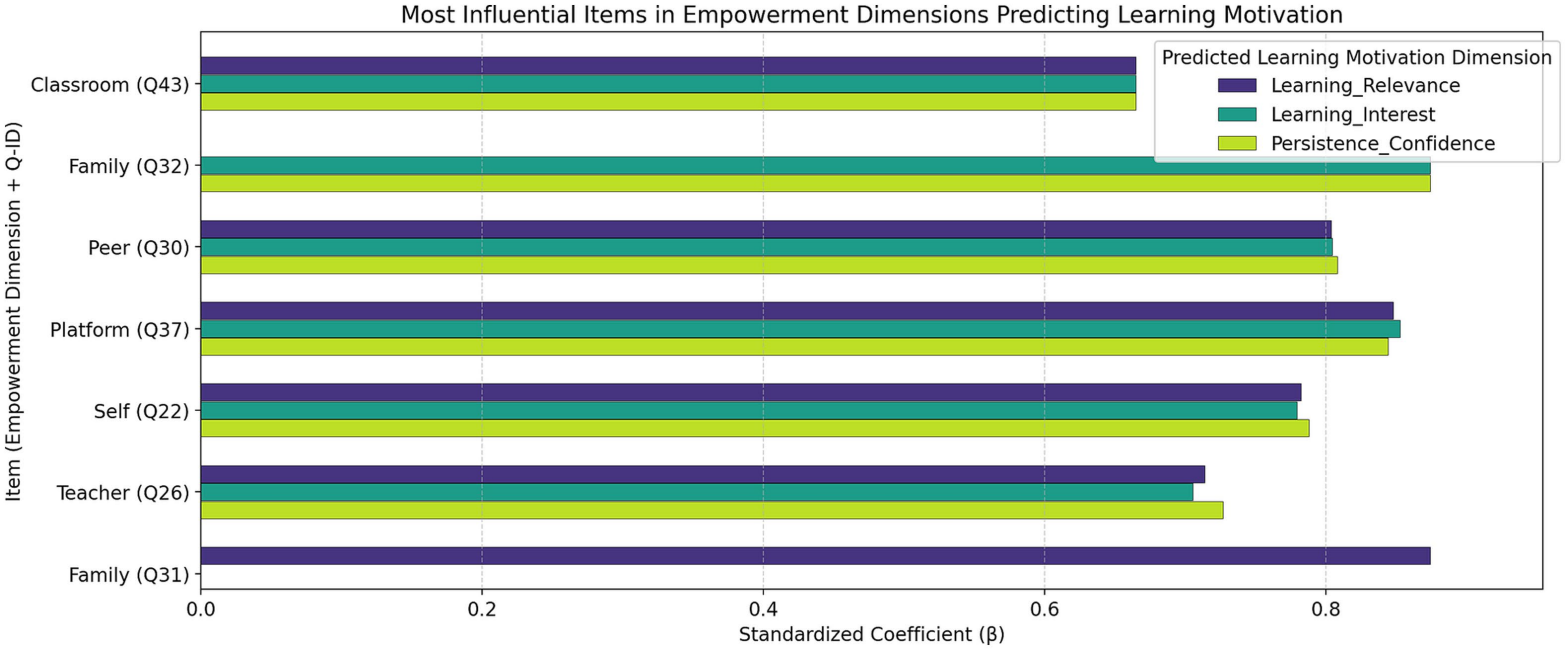
Most influential items within empowerment dimensions predicting learning motivation. Displayed are standardized *β* coefficients (*p* < 0.001) from multiple regression analyses across six empowerment dimensions.

To further identify specific factors with significant predictive power within each emotionally empowering dimension, this study constructed multiple linear regression models, using items from Q18–Q43 as independent variables and the three motivational subdimensions-“Learning Interest,” “Learning Relevance,” and “Persistence and Confidence”-as dependent variables. Items demonstrating both statistical significance (*p* < 0.05) and the largest standardized regression coefficients within each dimension were extracted as the most influential predictors (Figure 3).

Results indicate that the strongest item-level coefficients were observed for Family Empowerment (Q31/Q32) and Platform Empowerment (Q37), with comparatively smaller coefficients for Classroom Interaction (Q43). Specifically, the family empowerment items-Q31 (“Family members provide encouragement for my academic efforts”) and Q32 (“My family actively cares about my learning progress”)-showed the highest standardized coefficients (*β* ≈ 0.84–0.89, *p* < 0.001), suggesting that sustained family attention and emotional support play a pivotal role in enhancing students’ persistence and confidence. Similarly, the classroom empowerment item Q43 (“Classroom activities encourage interaction and active participation”) demonstrated more modest but still significant predictive power (*β* ≈ 0.66–0.67, *p* < 0.001), highlighting the importance of immersive and participatory learning environments in maintaining student engagement.

Platform Empowerment (Q37) also showed robust effects (*β* ≈ 0.83–0.85, *p* < 0.001), particularly on Learning Interest and Learning Relevance, underscoring that transparent progress tracking and feedback mechanisms provided by digital platforms can effectively strengthen students’ sense of achievement and task alignment. In contrast, Self-Empowerment (Q22) and Teacher Empowerment (Q26) demonstrated moderate-to-strong but significant influences (*β* ≈ 0.78–0.79, *p* < 0.001), confirming that intrinsic motivation and pedagogical scaffolding jointly contribute to sustained learning engagement. The Peer Empowerment (Q30) dimension exerted a positive and relatively strong effect (*β* ≈ 0.80–0.81), indicating that while peer collaboration facilitates social reinforcement, its motivational impact is slightly smaller than that of family and digital contexts.

In summary, different emotionally empowering pathways exert distinct predictive influences on students’ learning motivation. External affective supports-particularly family involvement and platform-related affordances-emerge as the most powerful predictors, while platform functionality and self-directed learning jointly sustain motivation through continuous feedback and autonomy support. These findings suggest that optimizing affective environments across both digital and interpersonal dimensions is critical for cultivating enduring learning motivation in digitally enhanced education. Given that item-level predictors were drawn from closely related self-report constructs, the large standardized β estimates may partially reflect shared method variance and measurement redundancy. Accordingly, these item-level coefficients are interpreted as highlighting *salient indicators within each facet* rather than precise estimates of independent causal effects.

### 3.5 Predictive effects of dual emotionally empowering pathways on learning interest, learning relevance, and persistence

To further investigate the specific effects of emotional value in digital teaching resources on learning motivation, this study established multiple linear regression models with “Platform Empowerment” and “Self-Empowerment” as core predictors, targeting the three motivational dimensions: “Learning Interest,” “Learning Relevance” and “Persistence and Confidence” (Table 2). Results demonstrated that both empowerment variables achieved statistical significance across all models, with excellent overall model fit. Notably, the highest explanatory power was observed for Learning Interest (*R*^2^ = 0.868). Although the models showed high explained variance (*R*^2^ = 0.830–0.868), such values may be inflated in cross-sectional self-report research when predictors and outcomes are conceptually proximal and measured within the same survey. Consistent with this concern, the Harman single-factor check indicated a strong general factor (first factor = 78.78% variance). Moreover, when all six empowerment dimensions were entered simultaneously as predictors, multicollinearity diagnostics indicated severe collinearity (VIF range: 123.2–308.1), suggesting substantial shared variance among empowerment facets. Therefore, we interpret the regression results primarily as evidence of strong *associations* and emphasize relative patterns across predictors rather than independent causal effects; as a robustness check under collinearity, ridge regression produced similar cross-validated explanatory performance (CV-R^2^ ≈ 0.860–0.877 across outcomes), supporting the stability of the overall predictive relationship.

**Table 2:** Multiple regression results for platform empowerment and self-empowerment predicting three dimensions of learning motivation.

Dependent variable	Platform empowerment	Platform empowerment_p	Self-empowerment	Self-empowerment _p	*R* ^2^
Learning_Interest	0.675626	7.198179e-178	0.269407	1.919696e-36	0.868067
Learning_Relevance	0.647395	1.917997e-134	0.293059	6.623509e-33	0.830315
Persistence and Confidence	0.631948	6.876747e-129	0.314588	3.363056e-37	0.830966

All coefficients are standardized *β*. All models significant at *p* < 0.001. Model fit indices: *R*^2^ represents the proportion of variance explained.

Specifically, platform empowerment demonstrated significant regression coefficients on all motivation dimensions: *β* = 0.676, *p* < 0.001 for “Learning Interest,” *β* = 0.647, *p* < 0.001 for “Learning Relevance,” *β* = 0.632, *p* < 0.001 for “Persistence and Confidence”. These robust effects underscore the critical role of the platform’s interface usability, feedback mechanisms and resource richness in initiating and sustaining student motivation. Concurrently, self-empowerment exerted significant though comparatively moderate influences across dimensions: *β* = 0.269, *p* < 0.001 on “Learning Interest,” *β* = 0.293, *p* < 0.001 on “Learning Relevance,” *β* = 0.315, *p* < 0.001 on “Persistence and Confidence”. This confirms that learners’ exploratory drive, self-regulatory capacity, and learning planning awareness constitute essential internal drivers for persistent academic engagement.

Furthermore, scatter plots of actual versus predicted values show that the prediction results of the three sets of models are highly consistent with students’ self-assessed motivation scores, with no systematic deviation observed, further validating the model’s stability and explanatory power. In summary, Platform Empowerment and Self-Empowerment, as highly operable affective support factors, play a critical role in enhancing students’ learning motivation. Future pedagogical design should focus on optimizing platform interaction experiences while cultivating students’ self-directed learning capabilities to systematically improve learning motivation and persistence.

## 4 Discussion

Grounded in SDT and Keller’s ARCS model of motivational design, this study investigates how emotionally empowering value embedded in digital teaching resources relates to learning motivation among five-year higher vocational medical students. By constructing a dual-dimensional “Platform-Self Empowerment” framework and adopting a tiered classification of learning motivation, this study helps clarify how these factors jointly relate to variations in motivational profiles.

First, motivation levels differed systematically across usage patterns and student characteristics. Students who engaged with digital resources more frequently showed higher motivation levels, consistent with prior evidence that sustained, meaningful use of digital resources is associated with stronger motivation and learning outcomes (Noor et al., 2022; Fatima et al., 2019). This pattern aligns with the “Relevance” and “Satisfaction” components of the revised ARCS model (Keller, 2010), suggesting that frequent engagement—especially when accompanied by timely feedback and clear task value—may help sustain motivational states (Chen et al., 2024; Zhu et al., 2022). In addition, motivation tended to decline with advancing grade levels, which may reflect increasing workload, novelty loss, or shifts in goal orientation across the training trajectory (Pettersson et al., 2024).

Second, regarding the dual-pathway framework, platform empowerment and self-empowerment were both significant predictors across motivational outcomes, with platform empowerment showing the stronger standardized effects in the regression models. This suggests that platform-level affordances (e.g., usability, feedback responsiveness, and resource richness) may function as proximal triggers of attention and relevance, whereas self-empowerment reflects internalized agency that supports persistence and confidence (Ganotice et al., 2023). Interpreted through SDT, external supports may create conditions for autonomy- and competence-supportive experiences, while internalized self-regulation helps maintain motivation when external stimulation fades.

Third, the item-level results highlight actionable design implications, although they should be read as salience indicators rather than independent causal effects. Items related to supportive feedback, emotional resonance, and goal clarity appeared particularly influential, consistent with the view that motivational internalization depends on perceived control and perceived value (Artino et al., 2012). Together, these findings suggest that emotionally empowering digital resources should prioritize transparent progress feedback, meaningful goal framing, and emotionally supportive learning cues, while also integrating supportive instructional and social environments to strengthen sustained engagement (Yu and Deng, 2022).

In summary, this study extends existing discussions on affective value in digital teaching resources by empirically linking emotionally empowering perceptions to multiple motivational outcomes within higher vocational education. Interpretation of effect magnitudes should be situated within the study’s measurement approach and cross-sectional survey design, while the observed patterns offer practical cues for prioritizing supportive features in digital resource design.

### 4.1 Research limitations and future directions

Despite certain advantages in theoretical modeling, variable design, and sample size, this study has the following limitations: First, the research sample was drawn from one institution using a hybrid cluster-convenience sampling strategy. Therefore, representativeness may be limited, and the instructor-mediated administration may introduce potential selection or social-desirability bias. Second, the cross-sectional design cannot yet reveal the dynamic evolution of learning motivation; moreover, as predictors and outcomes were obtained from a single self-report survey, common-method variance may inflate associations, and causal inference is not warranted. Furthermore, while current analyses mainly rely on linear regression and correlation testing, they have not systematically examined mediation-moderation pathways or structural model relationships; given the conceptual proximity among empowerment dimensions, multicollinearity and limited discriminant validity may affect the stability and interpretation of regression coefficients, and the relatively high R^2^ values should be interpreted with appropriate caution. To address these limitations, future studies should expand coverage and adopt more transparent sampling procedures, incorporate longitudinal tracking or experimental designs, integrate multi-source or multi-method data to reduce method-related inflation, and employ SEM with more thorough factor-analytic validation and complementary predictive modeling approaches for in-depth exploration.

## 5 Conclusion

This study proposed and tested a “Platform Empowerment–Self-Empowerment–Learning Motivation” framework grounded in SDT and the ARCS model, examining the associations between emotionally empowering value in digital teaching resources and motivation among five-year higher vocational medical students. Based on 1,303 valid questionnaires, motivation levels were relatively high overall but varied significantly across gender, grade, and usage frequency variables, with more frequent resource use associated with higher motivation tiers. Regression results indicated that both platform empowerment and self-empowerment were significant correlates of learning motivation, with platform empowerment showing stronger standardized effects in the dual-pathway models. These findings provide practical implications for digital resource optimization, suggesting that improving platform usability and feedback mechanisms and supporting learners’ autonomy and self-regulation may jointly strengthen motivation. Given the cross-sectional self-report design and the strong conceptual proximity among constructs, future research should adopt multi-source and longitudinal approaches to validate mechanisms and reduce potential inflation of associations.

## Data Availability

The raw data supporting the conclusions of this article will be made available by the authors, without undue reservation.
