# Supplementary material for: The impact of emotional value in digital teaching resources on learning motivation—an empirical analysis based on a questionnaire survey
Source: Front Psychol. 2026 Mar 24;17:1733324. doi: 10.3389/fpsyg.2026.1733324 (PMC13055621; doi:10.3389/fpsyg.2026.1733324)
Supplement: Supplementary file 1 [file Supplementary_File_1.docx]

**Supplementary Material 1**

**Questionnaire on the impact of emotional value on learning motivation in digital teaching resources**

Informed consent statement

Dear students:

Thank you for participating in this questionnaire survey! This questionnaire aims to study the impact of emotional value on learning motivation in digital teaching resources. Your answer will play an important role in the progress of this study. Please fill it out according to your actual situation.

This questionnaire is anonymous and all data will only be used for academic research. Please feel free to answer. Thank you for your support and cooperation!

Department of Medical Technology, Nantong Health College of Jiangsu Province

05 January 2025

**Section One: Demographic Profile and Digital Resource Usage**

Please fill in the following basic information

Q1.Your age:

Q2.Gender:

Q3Grade:

Q4.major:

Please evaluate the following statements based on your actual feelings and mark “√”.

Q5.Frequency of using digital teaching resources (such as APP, online courses, etc.):

A. Every day（ ）

B. 3-5 times a week（ ）

C. 1-2 times a week（ ）

D. 2-3 times a month（ ）

E. Occasionally（ ）

What are the types of digital teaching resources you are currently using (multiple selections are allowed, numbered by primary and secondary):

Q6. Online course platform (Xuexitong, Smart Education, etc.) ( )

Q7. Educational APP ( )

Q8. Electronic textbooks, courseware ( )

Q9. Teaching videos (such as learning videos on Bilibili) ( )

Q10. Others (please specify: _______)

What is your main purpose for using digital teaching resources? (Multiple choices are allowed):

Q11. Supplement classroom knowledge

Q12. Preview courses

Q13. Review and consolidate after class

Q14. Expand knowledge

Q15. Complete homework or tasks

#### Q16. Improve learning interest

#### Q17. Others (please specify: _______)

**Section Two: Emotional Value Empowerment Dimension Measurement**

**The following are questions about how digital teaching resources empower and influence your emotional experience in learning. Please rate the following statements based on your actual feelings (1-5), and please tick “√” in “☐”.**

（一）Self-reliance (personal emotional empowerment)

Q18.I am interested in the learning content itself and am willing to actively explore through resources.

☐Strongly disagree ☐Less agree ☐Generally **□**More agree ☐Strongly agree

Q19.The learning resources provide clear goals and planning tools, helping me manage my time and tasks effectively.

☐Strongly disagree ☐Less agree ☐Generally **□**More agree ☐Strongly agree

Q20.The design of the resources helps me overcome learning difficulties and boosts my confidence (e.g., through features like prompts and task breakdowns).

☐Strongly disagree ☐Less agree ☐Generally **□**More agree ☐Strongly agree

Q21.The gamified design of the resources (e.g., points and rewards) makes me more motivated and eager to complete learning tasks.

☐Strongly disagree ☐Less agree ☐Generally **□**More agree ☐Strongly agree

Q22.The diverse content (such as videos, texts, and interactive modules) helps me stay more focused on my learning goals.

☐Strongly disagree ☐Less agree ☐Generally **□**More agree ☐Strongly agree

**（二） Teacher empowerment (teacher emotional support)**

Q23.The tasks and questions designed by the teacher make learning feel interesting and challenging.

☐Strongly disagree ☐Less agree ☐Generally **□**More agree ☐Strongly agree

Q24.The feedback provided by the teacher through the resources (such as grades and suggestions) makes me feel recognized and encouraged.

☐Strongly disagree ☐Less agree ☐Generally **□**More agree ☐Strongly agree

Q25.The teacher's way of expression and interactive design make learning feel easy and enjoyable.

☐Strongly disagree ☐Less agree ☐Generally **□**More agree ☐Strongly agree

Q26.The multimedia content provided by the teacher (such as videos and case studies) makes it easier for me to understand and helps me remember more deeply.

☐Strongly disagree ☐Less agree ☐Generally **□**More agree ☐Strongly agree

**（三） Peer empowerment (social-emotional support)**

Q27.The sharing and collaboration with peers provide me with new learning perspectives and approaches to problem-solving.

☐Strongly disagree ☐Less agree ☐Generally **□**More agree ☐Strongly agree

Q 28.Working with peers to complete tasks gives me a sense of team support and belonging.

☐Strongly disagree ☐Less agree ☐Generally **□**More agree ☐Strongly agree

Q 29.Discussions with peers help me better understand the learning content and boost my confidence.

☐Strongly disagree ☐Less agree ☐Generally **□**More agree ☐Strongly agree

Q 30.Social features (such as comments and likes) make me feel the vibrancy and support of the community.

☐Strongly disagree ☐Less agree ☐Generally **□**More agree ☐Strongly agree

**（四） Family empowerment (supportive emotions)**

Q31.My family provides a quiet and comfortable environment for my learning and is willing to help solve technical issues (such as devices and internet access).

☐Strongly disagree ☐Less agree ☐Generally **□**More agree ☐Strongly agree

Q 32.The recognition and encouragement from my family make me feel valued and more motivated to achieve my learning goals.

☐Strongly disagree ☐Less agree ☐Generally **□**More agree ☐Strongly agree

Q 33.My family's active concern for my learning goals and progress enhances my confidence.

☐Strongly disagree ☐Less agree ☐Generally **□**More agree ☐Strongly agree

Q 34.Through using digital learning resources, parents can more intuitively understand the student’s learning process.

☐Strongly disagree ☐Less agree ☐Generally **□**More agree ☐Strongly agree

**（五） Platform empowerment (technical and design-based emotional support)**

Q35.The platform has a user-friendly and easy-to-navigate interface, which makes it convenient for me to use.

☐Strongly disagree ☐Less agree ☐Generally **□**More agree ☐Strongly agree

Q36.The platform provides a rich variety of resources that meet my diverse learning needs.

☐Strongly disagree ☐Less agree ☐Generally **□**More agree ☐Strongly agree

Q 37.The platform's learning progress tracking and feedback features make the learning process feel clear and well-organized.

☐Strongly disagree ☐Less agree ☐Generally **□**More agree ☐Strongly agree

Q 38.The platform runs smoothly, and the technical support is prompt, which makes me feel secure and efficient.

☐Strongly disagree ☐Less agree ☐Generally **□**More agree ☐Strongly agree

Q39.The platform's interactive and reward mechanisms (such as points and badges) enhance my enjoyment of learning and sense of achievement.

☐Strongly disagree ☐Less agree ☐Generally **□**More agree ☐Strongly agree

**（六）Classroom Interaction Empowerment (Situational and Process-based Emotional Support)**

Q40.Classroom interactions (such as discussions and task challenges) have increased my engagement and interest.

☐Strongly disagree ☐Less agree ☐Generally **□**More agree ☐Strongly agree

Q41.Classroom activities help me discover new ways to solve problems and understand the content more deeply.

☐Strongly disagree ☐Less agree ☐Generally **□**More agree ☐Strongly agree

Q42.The interactive sessions designed by the teacher make me feel the fun and value of the learning process.

☐Strongly disagree ☐Less agree ☐Generally **□**More agree ☐Strongly agree

Q43.Through classroom interactions, I am more willing to express my opinions and participate in discussions.

☐Strongly disagree ☐Less agree ☐Generally **□**More agree ☐Strongly agree

**Section Three: Assessment of Learning Motivation**

**Please evaluate the following statements.Based on your actual feelings, rate the following statements (1–5).Please mark “√” in the “☐”.**

**（一）Learning Interest and Attention (Attention)**

Q44.Learning resource content can stimulate my curiosity and sustain my attention.

☐Strongly disagree ☐Less agree ☐Generally **□**More agree ☐Strongly agree

Q45.The diverse designs in the resources (such as animations and case studies) make me willing to actively explore more content.

☐Strongly disagree ☐Less agree ☐Generally **□**More agree ☐Strongly agree

Q46.The novelty of the learning content gives me a sense of enjoyment in exploration.

☐Strongly disagree ☐Less agree ☐Generally **□**More agree ☐Strongly agree

**（二）Clarity and Relevance of Learning Goals (Relevance)**

Q47.Digital resources help me clearly understand the learning goals and closely relate them to my actual needs.

☐Strongly disagree ☐Less agree ☐Generally **□**More agree ☐Strongly agree

Q48.The content of the learning tasks is closely related to my life or professional background, which motivates me to complete them.

☐Strongly disagree ☐Less agree ☐Generally **□**More agree ☐Strongly agree

Q49.The current learning content has clear value for my future development (such as in career or academics).

☐Strongly disagree ☐Less agree ☐Generally **□**More agree ☐Strongly agree

**（三）Persistence in Learning and Self-Confidence**

Q50.When facing difficulties, the support provided by the resources helps me persist in completing learning tasks.

☐Strongly disagree ☐Less agree ☐Generally **□**More agree ☐Strongly agree

Q51.I believe that through effort I can master the learning content, and the feedback from the resources boosts my confidence.

☐Strongly disagree ☐Less agree ☐Generally **□**More agree ☐Strongly agree

Q52.The goals of the learning tasks are clear, and I know how to manage my time effectively to complete them.

☐Strongly disagree ☐Less agree ☐Generally **□**More agree ☐Strongly agree

Q53.I can reasonably plan my study time and strictly follow the schedule.

☐Strongly disagree ☐Less agree ☐Generally **□**More agree ☐Strongly agree

Q54.After completing learning tasks, I feel fulfilled and satisfied, and look forward to more challenges.

☐Strongly disagree ☐Less agree ☐Generally **□**More agree ☐Strongly agree

Q55.The design of the learning resources (such as rewards and interactive features) makes me feel the joy and sense of achievement in learning.

☐Strongly disagree ☐Less agree ☐Generally **□**More agree ☐Strongly agree

Q56.When I make progress or achieve success using digital teaching resources, it further strengthens my positive view of the resources and my willingness to use them.

☐Strongly disagree ☐Less agree ☐Generally **□**More agree ☐Strongly agree

**Supplementary Text 1**

Factor-analytic evidence and discriminant validity checks

To provide additional measurement evidence beyond Cronbach’s α, we conducted exploratory factor analyses (EFA) and discriminant validity checks for the empowerment and motivation measures. EFAs were performed separately for the empowerment items (Q18–Q43; 26 items) and motivation items (Q44–Q56; 13 items) using principal axis factoring (PAF) with an oblique rotation (Promax), given the theoretical expectation that dimensions are correlated. The number of factors retained was guided by theoretical structure, eigenvalues (>1), and scree-plot inspection. Factorability was excellent for both item sets (empowerment: KMO = 0.980; Bartlett’s χ²(325) = 56061.93, p < .001; motivation: KMO = 0.975; Bartlett’s χ²(78) = 30663.46, p < .001).

In the theory-consistent rotated solutions (six-factor for empowerment; three-factor for motivation), primary loadings were generally moderate-to-high (empowerment: approximately 0.53–0.81; motivation: approximately 0.64–0.77). Cross-loadings were observed for several items, indicating substantial shared variance among closely related facets—consistent with the conceptual proximity of emotionally empowering perceptions and motivational appraisals measured within the same survey.

Discriminant validity was further examined using the heterotrait–monotrait ratio (HTMT) across the nine constructs (six empowerment dimensions and three motivation subdimensions). HTMT values ranged from 0.807 to 0.980, with several comparisons exceeding conventional thresholds (e.g., >0.90), particularly among the motivation subdimensions and between certain empowerment facets and motivation outcomes. These results suggest that some constructs are highly overlapping, which may contribute to inflated association magnitudes and unusually high model explanatory power in regression analyses. Accordingly, we interpret effect sizes cautiously and emphasize relative patterns rather than independent causal effects. Future research may benefit from testing higher-order measurement structures (e.g., second-order factors consistent with an overarching emotionally empowering value construct) and incorporating multi-source or longitudinal measures to strengthen discriminant validation and reduce potential common-method inflation.
